# Functionally Defective High-Density Lipoprotein and Paraoxonase: A Couple for Endothelial Dysfunction in Atherosclerosis

**DOI:** 10.1155/2013/792090

**Published:** 2013-10-07

**Authors:** Esin Eren, Necat Yilmaz, Ozgur Aydin

**Affiliations:** ^1^Laboratory of Atatürk Hospital, 07040 Antalya, Turkey; ^2^Central Laboratories of Antalya Education and Research Hospital of Ministry of Health, 07100 Antalya, Turkey; ^3^Antalya Eğitim ve Araştırma Hastanesi Merkez Laboratuvarı Soğuksu, 07100 Antalya, Turkey

## Abstract

The endothelium is the primary target for biochemical or mechanical injuries caused by the putative risk factors of atherosclerosis. Endothelial dysfunction represents the ultimate link between atherosclerotic risk factors that promote atherosclerosis. HDL-C is thought to exert at least some parts of its antiatherogenic facilities via stimulating endothelial NO production, nearby inhibiting oxidative stress and inflammation. HDL-C is capable of opposing LDL's inductive effects and avoiding the ox-LDL's inhibition of eNOS. Paraoxonase 1 (PON1) is an HDL-associated enzyme esterase which appears to contribute to the antioxidant and antiatherosclerotic capabilities of HDL-C. “*Healthy HDL*,” namely the particle that contains the active Paraoxonase 1, has the power to suppress the formation of oxidized lipids. “*Dysfunctional HDL*,” on the contrary, has reduced Paraoxonase 1 enzyme activity and not only fails in its mission but also potentially leads to greater formation of oxidized lipids/lipoproteins to cause endothelial dysfunction. The association of HDL-C PON1 and endothelial dysfunction depends largely on the molecules with exact damaging effect on NO synthase coupling. Loss of nitric oxide bioavailability has a pivotal role in endothelial dysfunction preceding the appearance of atherosclerosis. Analyses of HDL-C and Paraoxonase1 would be more important in the diagnosis and treatment of atherosclerosis in the very near future.

## 1. Introduction

 Lipoproteins and their alterations are very frequently linked with increased risk of developing atherosclerotic disease. Recent studies introduce a function-based approach on evaluation of both the lipoprotein particles and the ultimate target: the endothelium. Considering particularly the high-density lipoprotein-cholesterol (HDL-C), there are two different definitions: HDL-C quantity, which means the circulating plasma levels of the particle; and HDL-C quality, which means the atheroprotective properties of HDL-C. Bare measurements of plasma concentrations, while shown to be epidemiologically predictive of atherosclerotic cardiovascular events in large populations, are insufficient to capture the functional variations in HDL-C particles and the risk of atherosclerotic disease associated with it. Functionality hypothesis suggests that measurement of HDL-C levels has no major relationship with how the HDL-C concentrations are being dynamically remodeled or the state of HDL-C capability [1].

The goal of this paper is to summarize the recent data on HDL-C functionality and to make connections between separate findings to figure out the full picture. We will particularly focus on HDL-associated antioxidant enzyme paraoxonase (PON1) and search for clues for the impact of HDL-C functionality on the “endothelial dysfunction.”

## 2. HDL-C and Atherosclerosis

HDL-C was isolated from animal serum in 1929 and introduced as a lipid rich “*α*-globulin” [1, 2]. Since then, HDL-C has been shown to have a variety of functions that contribute to its atheroprotective effects: promotion of macrophage cholesterol efflux, reverse cholesterol transport (RCT), antiinflammatory, antithrombotic, antiapopitotic, and antioxidative effects [1, 3, 4].

Lipoproteins are thought to play a pivotal role in the pathogenesis of atherosclerosis. There is a bulky literature on their exact behavior in the development of the disease, benefits in their measurement in diagnosis and followup of patients, and finally on their medical arrangements. The lipid-lowering statins have become very popular in the medical management of atherosclerosis. These drugs consistently and efficiently lower blood low-density lipoprotein-cholesterol (LDL-C) but decrease the risk of major cardiovascular events by 15% to 40% [2]. This substantial residual risk is the major force that shapes recent alternative therapeutic strategies. Researchers, finally, look beyond LDL-C and focus on HDL-C. The common sense states an inverse correlation between HDL-C blood levels and the risk of atherosclerosis [1]. Unlike LDL-C, HDL-C is in the mission to protect the artery wall from atherosclerosis.

## 3. Major Proteins of the HDL-C 

**Figure 1:**
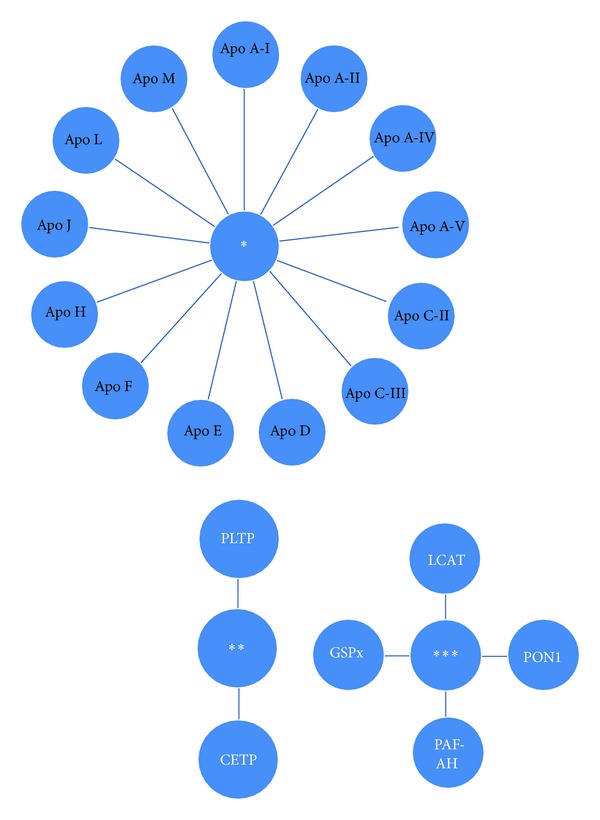
Proteins of HDL-C. *: apolipoproteins, **: lipid transfer proteins, ***: enzymes of the HDL-C proteome.

HDL-C constitutes a dynamic polydisperse group of particles which are central to lipid metabolism. The first HDL-associated protein moiety was identified in the late 1960s. By the early 1990s, HDL-C was thought to contain somewhere around fifteen proteins. Currently, up to two hundred individual proteins have been detected in human HDL-C samples. The presence of these proteins fits well into the general dogma of a primary HDL-C function as a lipid transport vehicle [1, 5–11]. HDL-C protein component is largely diverse, comprising structural apolipoproteins, enzymes, cofactors for enzymes, and numerous other proteins (Figure 1). While many HDL-C proteins fall within the general area of several biochemical pathways, numerous other functions are also present. The tremendous functional heterogeneity inherent to HDL-C is driven in large part by its compositional heterogeneity [9]. Many studies indicate that the HDL-C proteome can change in a variety of disease states and these changes are often related to at least *in vitro *measures of HDL-C function [9–11]. 

## 4. HDL-C Quantity versus HDL-C Quality

Epidemiological studies and prospective randomized trials have consistently shown a powerful inverse association between the magnitude of HDL-C and atherosclerosis. These observations reveal low plasma HDL-C levels to accompany accelerated atherosclerosis. However, some genetic syndromes that serve as an opportunity to test the outcomes of these studies present with conflicting results. They present with very low levels of HDL-C but are not associated with an increased risk of premature cardiovascular events. Patients with deficiency of plasma lecithin cholesterol acyltransferase (LCAT) enzyme have HDL-C concentrations lower than 0.40 mmol/L (15 mg/dL), but they do not show signs of an obvious increase in the risk of atherosclerosis. Mutations in the ABCA1 transporter gene, namely, Tangier disease, is an extreme situation in which HDL-C concentrations are generally undetectable and is not associated with the marked increase in atherosclerosis, expected from such a dramatic phenotype [5].

After all, reliability of the sole measurement of plasma HDL-C has been questioned in the determination of the risk of developing atherosclerotic disease [1]. Plasma HDL-C measurements fall short in predicting the functionality and composition of HDL-C, which we believe to be the key point that creates the contradiction in the literature. In recent studies, new definitions of HDL-C are frequently used: “*dysfunctional HDL,*” “*HDL dysfunction,” “HDL malformed,”* “*Healthy HDL,*” “*coronary artery disease HDL,”* “*chronic kidney disease HDL,”* “*obese HDL,”* and so forth [1, 6–8]. All in common, there is an emphasis on the functionality of HDL-C. The concept of “*HDL-dysfunction*” was introduced in the mind 1990s. The main proposition of “*dysfunctional*” or “*malformed*” was that, in some people, HDL-C was unable to do its duty properly [1]. HDL-C particles, which perform their biologic tasks are termed, “*functional HDL.*” If they do not, they are termed “*dysfunctional HDL*” [1]. So, the “*HDL hypothesis*” has begun to be replaced by the “*HDL function hypothesis,*” reflecting the growing consensus that measurement of one minor HDL-C component is far from the determination of the state of the particle [9].

## 5. Functions of the “*Healthy HDL*” 

The classical function of “*Healthy-HDL*” (H-HDL) is RCT. It removes cholesterol from peripheral tissues and delivers it to the liver or steroidogenic organs (*HDL-cargo*). The docking takes place as the major HDL-C apolipoprotein A-I (apoA-I) binds to the high affinity HDL-C soluble receptor-B1 (SR-BI) of the target tissue [12]. 

**Figure 2:**
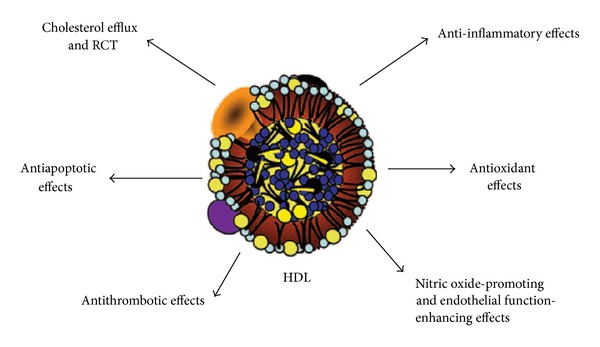
H-HDLs perform functions including several immunological activities.

H-HDL also has well-documented antioxidative properties. The antioxidant property of H-HDL is thought to be involved in potential antiatherogenic effects, but the exact mechanism is not known. H-HDL has been shown to prevent oxidative modification of LDL-C, thus reducing macrophage foam cell generation in the vessel wall [13]. Oxidized low-density lipoprotein (oxLDL) is the approved culprit in endothelial dysfunction. oxLDL induces endothelial damage, monocyte adhesion, and platelet aggregation and inhibits apoptosis and endothelial nitric oxide synthase (eNOS) expression/activity, all of which contribute to atherosclerotic process [14]. The other atheroprotective functions of H-HDL that have more recently attracted attention among other actions include its antiapopitotic, antithrombotic, and anti-infectious functions [1] (Figure 2). 

The Torcetrapib experience is the quintessential example of our inevitable failure, when the fair but complicated mechanisms of the human body are underestimated. Torcetrapib had been shown to substantially increase HDL-C concentrations by 50%–100%, promising an exuberant decrease in the development of atherosclerosis. However, the study was aborted due to unexpected consequences [1]. Elevations in blood pressure and aldosterone levels, which are not mechanism-situated effects, suggest that off-objective activities particular to Torcetrapib may have increased cardiovascular risk in study subjects [4]. Nevertheless, the Torcetrapib experience urged us to ask ourselves the crucial questions with answers that finally lead us to the contemporary concept of “*HDL functionality*” [1]. In our modern understanding, function of the garbage truck (HDL) is considered more important than its number in circulation.

Alternatively, if individual species of HDL-C performs distinct functions, it may be most advantageous to drug development strategies. The goal might be a raise in only certain ones to achieve benefits, particularly if altering other subspecies might have harmful effects on other important functions. These will be critical for determining alterations in the HDL-C proteome in the face of disease states such as atherosclerosis and other disorders. As stated previously, these studies will be useful for identifying new biomarkers for early diagnosis of disease, pinpointing new pathways for therapeutic mediation, and assessing the effectiveness of current and new therapeutics [9, 15].

## 6. Paraoxonase 1 and Atherosclerosis

Paraoxonase 1 (PON1) is an HDL-associated enzyme esterase which appears to contribute to the antioxidant and antiatherosclerotic capabilities of HDL-C [16]. Several prospective studies have shown that low PON1 is an independent risk factor for atherosclerotic events. Although this finding is not universal, low PON1 is a general feature of people who develop atherosclerosis [17, 18]. 

Briefly, PON1 possesses cardiovascular protective properties which result in the following antiatherogenic functions: [1] attenuated oxidative stress in serum, lipoproteins, macrophages, and atherosclerotic lesions; [2] decreased oxidized LDL-C uptake by macrophages; [3] inhibited macrophage cholesterol biosynthesis rate; [4] stimulated HDL-mediated cholesterol efflux from macrophages [19].

PON1 is synthesized in the liver and secreted into the bloodstream where it is capable of breaking down both man-made and naturally occurring compounds. Named for its ability to hydrolyze organophosphates like paraoxon found in insecticides, PON1 is also able to hydrolyze N-acyl-homoserine, a lactone used by pathogenic bacteria, and lipid peroxides, thereby inhibiting the formation of foam cells known to contribute to atherosclerosis [16, 20–22].

## 7. Tissues and PON's Gene Expression

The three PON genes (PON1, PON2, and PON3) are located adjacent to each other on chromosome 7 and share 65% similarity at the amino acid level. Liver is the principal tissue for PON1 gene expression. In 24 human tissues searched in a study, researchers detected PON1 expression in kidney and colon beside liver and fetal liver [20]. PON1 activity in human liver is primarily localized in microsomal fraction. The enzyme is orientated in the cell membrane before it is excreted to the serum and bound to HDL-C [20, 23]. 

PON2 is not detectable in serum. It is more widely expressed and is found in a number of tissues, including brain, liver, and kidney [24, 25]. PON2 enzyme resides in many tissues and is not released from the cells. It is located in the cell membrane with its active side exposed to the outer side of the cell. 

HDL-C has the ability to inactivate oxidized phospholipids. This ability is in part related to PON1 activity, but other enzymes or properties of HDL-C are also included. Interestingly, factors changing the PON1 activity like genetic, pathological, physiological, pharmacological, and lifestyle can similarly change the levels of HDL-C [1, 26] (Tables 1, 2, and 3).

Several transcription factors and genes related to oxidative stress were also identified and could play substantial roles in the regulation of PON1 concentration in the blood or the inactivation of PON1 which would decrease activity even in the presence of high levels of the enzyme (such as diabetes) [26, 27].

The heritability of paraoxonase activity varies with substrate, *h* ^2^ = 0.65 for phenyl acetate (PON1-ARE), *h* ^2^ = 0.73 for paraoxon (PON1), and *h* ^2^ = 0.79 for dihydrocoumarin (PON1-lact), after the inclusion of sex and age in the model. Cumulatively, age, sex, and their interaction effect explain less than 4% of the variation in PON1 activity for all substrates [27]. Although the PON region explains a large degree of the variation in PON1 activity, blood PON1 activity levels are still better predictors of disease than PON1 genotypes alone. There is at least a 40-fold variation in serum PON1 activity among individuals; while a portion of this variation is explained by genetic polymorphisms, the possible impress of exogenous factors also needs to be taken into account. Just as this statement focuses on factors which may increase PON1 activity and/or expression, one should not ignore that life style may decrease PON1 activity [28].

The effects of inhibition by different heavy metals on the immobilized and free paraoxonase enzyme activities indicated that different inhibitors exhibit different inhibition patterns (competitive, noncompetitive, and mixed). The pretreatment of human test sample with dithiothreitol (DTT) protects against the inhibitory effect of mercurials. These results confirmed the essential role of the –SH groups to maintain the catalytic activity of PON1 and suggest the existence of two types of –SH groups that could differ in their localization [26–30].

## 8. “*Adapted*” and “*Ancestral*” Substrates of PON1

Paraoxonase—so named because of its ability to hydrolyze the toxic metabolite of parathion, paraoxon—was also shown early after its identification to manifest arylesterase (ARE) activity. The ability to hydrolyze paraoxon was employed in the 1960s as the method to measure PON1 activity in several species and tissues. Hence, research on PON1 function was focused on trying to distinguish the native or “*ancestral*” function of this enzyme from all other secondary or “*adapted*” functions. Although the preferred endogenous substrate of PON1 remains unknown, lactones (especially Hcy-thiolactone) comprise one possible candidate class. PON1 has also been shown to metabolize a number of drugs and prodrugs via its lactonase activity (PON1-lact). PON1 exerts a protective effect against oxidative damage of cells and lipoproteins and modulates the susceptibility of HDL-C and LDL-C to atherogenic modifications such as homocysteinylation [1, 16].

## 9. Antioxidant Activity HDL-Associated Enzyme PON1

After the introduction of the oxidative stress hypothesis of atherosclerosis and the discovery of antioxidant effect of HDL-C, PON1 attracted significant interest as a protein that is responsible for the most of antioxidant properties of HDL-C [1]. Experimental studies have indicated that impaired PON1 activity leads to “*dysfunctional HDL”*. Experimental animal studies provide strong evidence that PON1 is required to enable HDL's antioxidant properties. In animal model studies, an accelerated development of atherosclerosis was shown in PON1 deficient mice [46]. Vice versa, overexpression of human PON1 resulted in a reduced atherosclerotic lesion formation in mice, further suggesting that reduced paraoxonase activity may contribute to the development of atherosclerosis. Reduced PON1 activity has been observed to be a substantially better predictor of atherosclerotic risk, compared to functional PON1 genetic variants in patients undergoing coronary angiography, suggesting that reduced PON1 activity is associated with a more rapid progression of atherosclerotic vascular disease [6, 46, 47].

The exact antioxidant mechanism of PON1 is still unknown. Incubation of purified PON1 with hydrogen peroxide or lipid peroxides partly decomposes them. PON1 is especially effective in the decomposition of linoleate hydroperoxides. Existence of an enzymatic mechanism is supported by the observation that heat inactivation of purified PON1 abolishes its antioxidant effect. PON1 may be interacting with apoA-I and LCAT to inhibit LDL-C oxidation, with the combination preventing LCAT inactivation. Purified PON1 protects HDL-C and LDL-C from oxidation catalyzed by copper ions [16, 20].

## 10. HDL-C and Endothelial Function 

The endothelium is a primary target for mechanical and biochemical injuries caused by putative risk factors of atherosclerosis [48]. It has traditionally been considered as an inert component of the vessel wall, but silent endothelium produces NO, which acts to inhibit cellular pathways of inflammation, proliferation, and thrombosis [49, 50]. HDL-C is thought to exert part of its antiatherogenic effect by stimulating endothelial NO production and inhibiting oxidant stress and inflammation [10, 51]. In vascular smooth muscles, HDL-C acts proinflammatory, promigratory, and degradative actions on endothelium and platelets [12]. Therefore, by modulating the production/activity of a variety of endothelium-derived factors, such as NO, PGI_2_, PAF, and vWF, HDL-C may affect both vascular tone and thrombogenicity [49].

Studies have clearly demonstrated the ability of HDL-C added to endothelial cells in culture to significantly enhance eNOS activity in a manner that is dependent on SR-BI [12, 50]. HDL-C induces eNOS and PON-1 activities and maintains vascular health. Several studies demonstrated that HDL-C interaction with SR-BI modifies endothelial cell membrane lipid distribution and morphology, thus potentially influencing eNOS activity [48]. Moreover, HDL-C can oppose LDL-C induction of platelet aggregation, serotonin release, and thromboxane B2 production and can block ox-LDL inhibition of eNOS [1, 52, 53]. 

SR-BI appears as a major player in HDL-induced vasodilation, mediating the production of another potent vasodilator, PGI_2_. HDL-C can also regulate the expression of COX-2 and PGI-2 release in endothelial cells to exert antiatherogenic functions [51]. Incubation of cultured endothelial cells with HDL-C causes a dose-dependent increase of PGI_2_ release, which is prevented by a COX inhibitor, implying an effect on PGI_2_ synthesis. It was hypothesized that HDL-C enhances COX-2 expression through NF-*κ*B activation. It is well known that S1P, the phospholipid content of HDL-C, binding to S1P receptors can increase COX-2 expression and PGI-2 release through p38MAPK/CREB pathway [54–57]. Recently Liu et al. reported that apoA-I induces COX-2 expression and PGI-2 release through ABCA1 and the actuation of intracellular p38 MAPK and ERK1/2, as well as JAK2 pathways, and apoA-I can reinforce these effects with S1P in human umbilical vein endothelial cell. These novel effects of apoA-I could in part mediate antiatherogenic effects of HDL-C [55].

ET-1 is a potent vasoconstrictor, and its elevation is considered a prognostic marker in patients with atherosclerosis. It binds to specific G protein-coupled receptors on smooth muscle cells to reverse the response to NO. ET-1 is highly expressed in damaged vessels. The vasodilating peptides (atrial natriuretic peptide, brain natriuretic peptide and adrenomedullin) perform their action not only by merely opposing the effects of ET-1 but also by inhibiting ET-1 production [58]. HDL-C similarly inhibits the secretion of ET-1. So, HDL-C may indeed prevent the vasoconstrictor effects of ET-1. Also, HDL-C inhibits vascular endothelial inflammation by increasing 3*β*-hydroxysteroid-Δ24 reductase expression and inducing heme oxygenase-1 [59]. 

HDL-C also has diverse antiinflammatory actions in endothelial cells. PAF-AH is an antioxidant enzyme preventing LDL-C oxidation by hydrolysis of oxidized phospholipids [12, 60]. Human plasma PAF-AH is in part associated with HDL-C. Therefore, by limiting PAF production by endothelial cells and enhancing its degradation by circulating enzymes, HDL-C may avoid PAF-induced adhesion of leukocytes to the activated endothelium, which may well contribute to the antiadhesive effects of HDL-C [48]. Oxidatively fragmented phospholipids with PAF-like activity generated by endothelial cells exposed to lipid-soluble hydroperoxides are released into the surrounding medium, in contrast to PAF, synthesized by endothelial cells in response to physiologic mediators which remain focal [61]. HDL-C enhances NO and PGI_2_ production and limits PAF activity [62]. 

Von Willebrand factor (vWF) is another protein expressed by endothelial cells that plays an essential role in platelet adhesion and aggregation. The blood vWF concentrations are countercorrelated with plasma HDL-C, suggesting that HDL-C may inhibit vWF production [63]. Incubation of cultured bovine aortic endothelial cells with HDL-C enhances endothelial cell immigration. This effect is specific and comparable to that of basic fibroblast growth factor, the prototypical agonist of endothelial cell movement [48, 63]. 

In endothelial cells and their progenitors, HDL-C retards apoptosis and stimulates proliferation and migration [64]. Initially, it was proposed that the HDL-induced proliferation occurs through a protein kinase C-mediated pathway. HDL-C apolipoproteins were required for this effect. Inflammatory cytokines, products of lipid peroxidation, and growth factor lack are potent apoptotic stimuli for endothelial cells [65, 66]. HDL-C protects cultured human endothelial cells from TNF-*α*-induced apoptosis. The apolipoprotein composition of HDL-C affects the antiapoptotic activity, with apoA-I-containing particles being the most effective [48, 65–67].

## 11. HDL-C Functionality and Endothelial Dysfunction

Injury to the endothelium gives way to several alterations of endothelial physiology, namely, endothelial dysfunction [68]. Endothelial dysfunction represents a link between atherosclerotic risk factors promoting atherosclerosis [69]. So, the noninvasive measurement of endothelial function is a focus of interest to assess atherosclerotic disease. 

 HDL-C has been shown to promote endothelial generation of NO *in vitro* and improve endothelial function and arterial vasoreactivity *in vivo*, providing another potential antiatherogenic mechanism and a basis for assessing the effects of HDL-targeted therapies. 

Assaying endothelial NO production in response to HDL-C could provide the basis of an *in vitro* proxy of endothelial function and permit assessment of this potentially important function of HDL-C. In principle, production of nitrite and nitrate is generated by incubating endothelial cells with L-arginine, the substrate of nitric oxide synthase (eNOS) [50, 70].

Oxidized LDLs are potent inducers of endothelial dysfunction. Protective effects of HDL-C on endothelial function are quite likely due to their capacity to counteract the effects of oxidized LDL-C [1, 12]. A low plasma HDL-C concentration is an independent predictor of endothelial dysfunction in healthy individuals and atherosclerotic patients [71]. 

A decrease in NO bioavailability is a prominent feature of endothelial dysfunction. Injury to vascular endothelium induces the expression of cell adhesion molecules (CAMs), such as vascular cell adhesion molecule-1, intercellular adhesion molecule-1, E-selectin, and P-selectin [48, 71]. HDL-C downregulates TNF-*α*-induced CAM expression in endothelial cells. Finally, the inhibition of CAM expression by HDL-C results in a significant reduction of leukocyte adhesion to cultured endothelial cells [48, 72]. Recently, Speer et al. reported that HDL-CKD reduced endothelial NO availability via toll-like receptor-2 (TLR-2), leading to impaired endothelial repair [73]. It is shown that mutations in apoA-I, ABCA1, and LCAT have lead effect on the antioxidant/anti-inflammatory properties of HDL-C [57]. Chronic inflammation, altering the lipid moiety of high-density lipoprotein, dramatically changes H-HDL to dysfunctional-HDL. The hypothesis that HDL-C is rendered dysfunctional in a disease was further supported by the observation that, despite the high levels of HDL-C, coronary artery disease patients had less antioxidative activity as measured by inhibition of phospholipid oxidation [74].

## 12. PON1 and Endothelial Dysfunction 

PON1 is directly involved in the pathogenesis of atherosclerosis by the modulation of NO bioavailability. Many potential mechanisms have been proposed for HDL-associated PON1's antiatherogenic effects, including the capability of HDL-C to inhibit inflammation and regulate NO production by endothelial cells. HDL-C from healthy individuals causes an increase in bioavailable eNO, while HDL-C from patients with atherosclerotic diseases causes no increase or an actual decrease in eNO [47]. H-HDL from healthy individuals activates the production of the antiatherosclerotic and anti-thrombotic signaling molecule NO by eNOS; it blunts adhesion molecule expression, attenuates tissue factor and E-selectin expression, and accelerates endothelial cell migration, so optimizing endothelial repair and the totality of the intimal layer [48, 75–77]. 

Normally, “*healthy-HDL*” includes active PON1, which suppresses the formation of oxidized lipids and lipoproteins such as MDA [6]. “*Dysfunctional-HDL*” has reduced PON1 activity that potentially leads to greater formation of MDA, which activates lectin-like oxidized LDL receptor-1 (LOX 1) and thereby stimulates PKC*β* [6, 78]. The LOX-1 is an oxLDL receptor expressed in vascular endothelium and a multiligand receptor implicated in endothelial dysfunction and atherosclerosis [31, 32]. 

Recently, Besler et al. demonstrated that, through a course involving the endothelial LOX-1, “*dysfunctional-HDL*” actuates endothelial PKC*β*II, which in turn inhibits Akt-activating phosphorylation and eNOS-activating phosphorylation events and eNO production. Also, recognizing that endothelial LOX-1 is activated by oxidized lipids, they then appreciated the potential role of MDA and found that MDA content is increased in “*dysfunctional-HDL*” compared with “*healthy-HDL*” [46, 47, 78]. At this point, it should be remembered that HDL-associated PON1 enzyme critically inhibits these lipids oxidations [1]. Actually, Besler et al. importantly indicate that HDL-associated PON1 enzyme activity has a major impact on endothelial function, which is consistent with the recorded inverse relationship between PON1 activity and atherosclerotic disease development [47].

It is unclear what causes the downregulation of PON1 activity in “dysfunctional-HDL-C,” even though its abundance is increased. It is also unknown whether the loss in PON1 enzyme activity leads to alterations in other HDL-C constituents besides MDA that activates LOX-1 [47]. 

Apart from the expression of PON1 in the liver, PON1's liberation into the circulation represents a key step in the modulation of its circulating concentration and activity. In this meaning, the suggested mechanism by which PON1 would be liberated has been suggested to involve SR-BI [41]. eNOS activation by HDL-C entails apoA-I-dependent binding of the lipoprotein to SR-BI in endothelial cells; this causes cholesterol efflux that is sensed by SR-BI and begins a signaling cascade including the activation of Src kinases, PI3K, and Akt, which phosphorylates eNOS at Ser1177 to enhance eNOS activity [48, 79]. These courses are dependent on the adaptor protein PDZK1, which binds to the extreme C terminus of SR-BI. 

Indeed, SR-BI, Src, and PI3K dependent Erk MAPK activation is also required for eNOS activation by HDL-C. In addition, HDL-associated S1P and related molecules may activate the lysophospholipid receptor S1P3 to stimulate eNOS [48]. Recently, Matsuo et al. reported that “HDL-obese” mediated a reduced eNOS-Ser1177 phosphorylation, whereas a significantly increased eNOS-Thr495 phosphorylation when compared to “H-HDL” [80]. In an experimental study, administering small apolipoprotein-mimetic peptides (Apo-A1) to mice also reduced atherogenesis [76].

Interestingly, factors modulating the HDL-associated PON1 enzyme activity are the same for endothelial modulation, encouraging the proposal that HDL-associated PON1 enzyme activity is the cornerstone for endothelial function.

The findings suggest that assays of HDL-associated PON1 enzyme action on endothelium may increase our knowledge to assign atherosclerotic disease risk, and they may boost our understanding of the outcomes of future trials testing HDL-associated PON1 enzyme targeted therapies. 

## Figures and Tables

**Table 1:** There are several transcription factors and pathways that regulate PON1 expression.

Some important PON1 regulator factors	Modulator signal	
High glucose level	Protein kinase C (PKC)	↑
High insulin level	Protein kinase C (PKC) Zeta (PKC *ζ*)	↑
Statins	p44/p42 MAP	↑
Fibrates	SREBP-2, PPAR**α**	↑
Rosiglitazone	PPAR**γ**	↑
Aspirin (salicylate)	AhR	↑
Dietary polyphenols	AhR	↑
Quercetin	SREPB-2	↑
Pomegranate	PKA, PPAR-*γ*	↑
*β*-Carotene	p-AMPK	↑
Cholesterol lowering alkaloid (benzyl tetrahydroquinoline)	JNK	↑
Eplerenone	Aldosterone	↑
Bile acids	FXR, FGFR4	↓
Urokinase-type plasminogen activator (uPA)	PPAR**γ**	↓
LPS and inflammatory cytokines	PPAR**δ**	↓
Testosterone	?	↑
Estrogen and methoxyprogesterone acetate		
Erythropoietin beta		
Ethanol		
Light drinkers		↑
Heavy drinkers		↓

**Table 2:** Major activators and inactivators of PON1.

Major PON1 inactivators	Reversal effect by
Smoking	Free thiols (GSH, L-cysteine, etc.)
Oxidative stress	Antioxidants (vitamin E, Carotenoids, Flavonoids)
High cholesterol	Statins
High triglycerides	Fibrates
High glucose	Insulin
High fructose	Insulin
Atropine	
Ampicillin	
Ciprofloxacin	
Clindamycin sulfate	
Oral contraceptives	
Copper, zinc, mercury	
Manganese, cobalt, cadmium	

**Table 3:** Serum PON1 enzyme activity and concentration have also been shown to be modulated by lifestyle and dietary factors.

Modulators (dietary and lifestyle)	∗
Exercise [31, 32]	↑
Olive oil consumption [33, 34]	↑
Green tea consumption [34]	↑
Pomegranate juice [35, 36]	↑
Meal frequency [37]	↓
Fasting [38, 39]	↑
High fat diet [40]	↓
Hormone replacement [41]	↑
Mediterranean diet [42]	↑
Soy isoflavones [43]	↑
Beta-carotene [44]	↑
Light ethanol drinkers	↑
Heavy ethanol drinkers [45]	↓

*PON1 activation and concentration.

## Abbreviations

apoA-I: Apolipoprotein A-I
ARE: Arylesterase
CAMs: Cell adhesion molecules
CETP: Cholesteryl ester transfer protein
ET-1: Endothelin 1
GSPx: Glutathione selenoperoxidase
HDL-C: High-density lipoprotein-cholesterol
H-HDL: Healthy-HDL
LCAT: Lecithin cholesterol acyltransferase
LDL-C: Low-density lipoprotein-cholesterol
oxLDL: Oxidized low-density lipoprotein
LOX 1: Lectin-like oxidized LDL receptor-1
NO: Nitric oxide
eNOS: Nitric oxide synthase
PAF: Platelet-activating factor
PLTP: Phospholipid transfer protein
PON1: Paraoxonase 1
SR-BI: HDL soluble receptor-B1
TLR-2: Toll-like receptor-2
TNF-*α*: Tumor necrosis factor alpha.
